# A rapid analysis of plasma/serum ethylene and propylene glycol by headspace gas chromatography

**DOI:** 10.1186/2193-1801-2-203

**Published:** 2013-05-01

**Authors:** Alexandra Ehlers, Cory Morris, Matthew D Krasowski

**Affiliations:** Clinical Chemistry Laboratory, Department of Pathology, University of Iowa Hospitals and Clinics, Iowa City, IA 52242 USA

**Keywords:** Ethylene glycol, Glycols, Gas chromatography, Propylene glycol, Toxicology

## Abstract

A rapid headspace-gas chromatography (HS-GC) method was developed for the analysis of ethylene glycol and propylene glycol in plasma and serum specimens using 1,3-propanediol as the internal standard. The method employed a single-step derivitization using phenylboronic acid, was linear to 200 mg/dL and had a lower limit of quantitation of 1 mg/dL suitable for clinical analyses. The analytical method described allows for laboratories with HS-GC instrumentation to analyze ethanol, methanol, isopropanol, ethylene glycol, and propylene glycol on a single instrument with rapid switch-over from alcohols to glycols analysis. In addition to the novel HS-GC method, a retrospective analysis of patient specimens containing ethylene glycol and propylene glycol was also described. A total of 36 patients ingested ethylene glycol, including 3 patients who presented with two separate admissions for ethylene glycol toxicity. Laboratory studies on presentation to hospital for these patients showed both osmolal and anion gap in 13 patients, osmolal but not anion gap in 13 patients, anion but not osmolal gap in 8 patients, and 1 patient with neither an osmolal nor anion gap. Acidosis on arterial blood gas was present in 13 cases. Only one fatality was seen; this was a patient with initial serum ethylene glycol concentration of 1282 mg/dL who died on third day of hospitalization. Propylene glycol was common in patients being managed for toxic ingestions, and was often attributed to iatrogenic administration of propylene glycol-containing medications such as activated charcoal and intravenous lorazepam. In six patients, propylene glycol contributed to an abnormally high osmolal gap. The common presence of propylene glycol in hospitalized patients emphasizes the importance of being able to identify both ethylene glycol and propylene glycol by chromatographic methods.

**Electronic supplementary material:**

The online version of this article (doi:10.1186/2193-1801-2-203) contains supplementary material, which is available to authorized users.

## Introduction

Consumption of ethylene glycol continues to be a public health problem (Kraut & Kurtz 2008). Ethylene glycol is most commonly found in automobile antifreeze. Ethylene glycol is metabolized by a series of steps to glycolic acid, glyoxylic acid, and finally oxalic acid, the latter with the potential to cause severe renal injury (Barceloux et al. 1999; Jammalamadaka & Raissi 2010). The definitive laboratory method for detection and quantitation of ethylene glycol in the serum/plasma is gas chromatography (GC), preferably with mass spectrometric detection (GC/MS) (Wu et al. 1995). Rapid enzymatic assays for ethylene glycol are available and have been used in the veterinary setting (Malandain & Cano 1996). However, these rapid assays suffer from lack of specificity, particularly cross-reactivity with chemically related compounds such as propylene glycol and 2,3-butanediol, although recent progress has been made in improving specificity (Juenke et al. 2011). Indirect measures of ethylene glycol ingestion include osmolal and anion gap, both of which may be elevated in ethylene glycol and methanol ingestions (Krasowski et al. 2012; Lynd et al. 2008). However, osmolal gap elevation is not specific to toxic alcohol or glycol ingestion, with osmolal gap elevations seen in diabetic ketoacidosis, alcohol ketoacidosis, renal failure, shock, and recent mannitol infusion (Krasowski et al. 2012; Lynd et al. 2008;Almaghamsi & Yeung 1997; Braden et al. 1993; Dursun et al. 2007; Garcia-Morales et al. 2004; Gill et al. 2005; Guglielminotti et al. 2002; Huff 1990; Sklar & Linas 1983).

Propylene glycol is chemically similar to ethylene glycol and is also used in some automobile antifreezes (Kraut & Kurtz 2008; Zar et al. 2007). Propylene glycol is generally much less toxic than ethylene glycol and is found in a variety of products including beverages, cosmetics, ointments, activated charcoal preparations, and as a diluent for intravenous preparations of poorly water-soluble drugs such as diazepam, etomidate, and lorazepam. Propylene glycol toxicity has been described in overdoses of propylene glycol-containing antifreeze (Brooks & Wallace 2002). A number of studies have detailed propylene glycol toxicity from repeated intravenous administrations of medications containing propylene glycol as the diluent, particularly lorazepam used for extended sedation (e.g., intubated patients on mechanical ventilation) (Al-Khafaji et al. 2002; Arbour 1999; Chicella et al. 2002; Parker et al. 2002;Wilson et al. 2005).

In this study, we describe a rapid HS-GC method for simultaneous quantitation of ethylene glycol and propylene glycol in human serum and plasma samples. Previous studies have reported GC (Balikova & Kohlicek 1988;Houze et al. 1993; Porter & Auansakul 1982; Smith 1984) methods with liquid injections for ethylene glycol. More recently, a GC-MS method has been described for ethylene glycol determination (Porter & Rutter 2010); however, many clinical laboratories do not have access to GC-MS instrumentation. The method we describe utilizes HS-GC instrumentation commonly used for measurement of ethanol, methanol, acetone, and isopropanol (‘toxic alcohols’) and allows for HS-GC for the toxic alcohols, ethylene glycol, and propylene glycol. The relatively high boiling points of ethylene glycol (197°C) and propylene glycol (188°C) necessitate derivatization prior to HS-GC. We utilize a simple derivitization step with phenylboronic acid prior to HS-GC, adapting from the original phenylboronic acid GC liquid injection method (Porter & Auansakul 1982). We also present a retrospective analysis into determination of ethylene glycol and propylene glycol in specimens at an academic medical center central clinical laboratory.

## Experimental

### Reagents and materials

Ethylene glycol was obtained from Fisher Scientific (Pittsburgh, PA, USA). 1,2-Propanediol (propylene glycol), 1,3-propanediol, acetone (HPLC grade), 2,3-butanediol, diethylene glycol, and phenylboronic acid were all obtained from Sigma-Aldrich (St Louis, MO, USA). Lyphochek Drug Free Serum was obtained from Bio-Rad (Hercules, CA, USA). HPLC grade deionized water was prepared in-house.

A glycol stock standard was prepared to contain ethylene glycol and propylene glycol both at a concentration of 1000 mg/dL in drug free serum. Additional working standards were prepared by diluting the stock standard with drug free serum to obtain concentrations of 200 mg/dL, 100 mg/dL, 50 mg/dL and 10 mg/dL. An internal standard solution was prepared by adding 75 μL of 1,3-propanediol to 100 mL HPLC grade deionized water (concentration ~79.5 mg/dL). A 5 mg/mL solution of phenylboronic acid was prepared in HPLC grade acetone.

### HS-GC analysis

The GC system consisted of a PerkinElmer (Waltham, MA, USA) Clarus 580 GC with a PerkinElmer TurboMatrix 40 headspace sampler. The Clarus 580 was equipped with a flame ionization detector and an Elite 200 capillary column (PerkinElmer). To 50 μL of sample (standard, control, or patient sample) in a small TDX centrifuge tube, 50 μL of the 1,3-propanediol internal standard solution, and 200 μL of the phenylboronic acid in acetone solution were added. The samples were vortexed for 5 seconds and centrifuged at 13,200 RPM for 1 minute to remove precipitated proteins. 10 μL of the supernatant was transferred to a headspace vial, sealed, and placed on the TurboMatrix 40. Vials were thermostatted for 9 minutes in a 140°C oven before injecting for 0.02 minutes onto the GC for analysis. The headspace needle and transfer line were at 180°C and 205°C, respectively, with the transfer line pressure set at 40 psi.

The GC oven was temperature programmed with an initial temperature of 80°C, increasing to 120°C at a rate of 20°C/minute, followed by an increase to 300°C at a rate of 45°C/minute with a hold at 300°C for 0.5 minutes. The temperature program was extensively optimized to decrease analysis time while maintaining good peak separation and preventing contamination of the column to allow detection of toxic alcohols (ethanol, methanol, isopropanol) on the same column. GC injector and detector temperatures were both set at 250°C. The method could be switched from toxic alcohol to glycols analysis within 2 minutes.

### Retrospective analysis

We have previously presented a large retrospective study of toxic alcohol and glycol analysis in patient samples at an academic medical center (Krasowski et al. 2012). In this study, we focus on the subset of 153 patients where GC analysis for ethylene glycol and propylene glycol was performed. Osmolal gap was calculated using a formula by Khajuria and Krahn (2005): osmolal gap = (Measured osmolality) – {2 x [Sodium] + (1.15 * [Glucose]/18) + ([BUN]/2.8) + (1.2 * [ETOH]/4.6), where [Sodium] is plasma sodium concentration in mEq/L, [Glucose] is plasma glucose concentration in mg/dL, [BUN] is plasma blood urea nitrogen in mg/dL, and [ETOH] is plasma ethanol concentration in mg/dL. Anion gap was equal to the plasma sodium concentration minus the sum of plasma bicarbonate and chloride concentrations (all measured in mEq/L). At the medical center clinical laboratory, 15 or greater was considered abnormal for both anion and osmolal gaps. All laboratory measurements were performed in the central Clinical Chemistry laboratory. Serum/plasma electrolytes, BUN, glucose, and ethanol were determined on high volume chemistry analyzers (Roche P modules, Roche Diagnostics, Inc., Indianapolis, IN, USA). Serum/plasma osmolality was determined by freezing point depression (Model 2020 osmometer, Advanced Instruments, Inc., Norwood, MA, USA). The project had Institutional Review Board approval from the University of Iowa.

## Results and discussion

**Figure 1:**
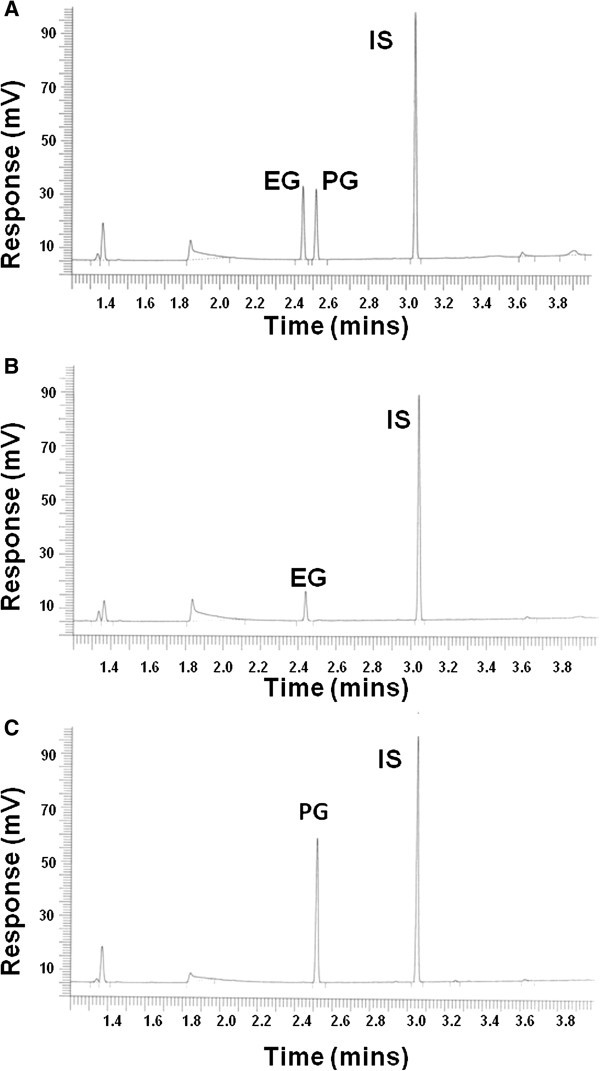
**HS-GC separation of the phenylboronic derivatives of ethylene glycol (EG) and propylene glycol (PG) with 1,3-propanediol as the internal standard (IS).** (**A**) Quality control plasma sample containing 25 mg/dL each of EG and PG. (**B**) Patient plasma sample containing 11.6 mg/dL of EG. (**C**) Patient plasma sample containing 53.4 mg/dL of PG.

**Figure 2:**
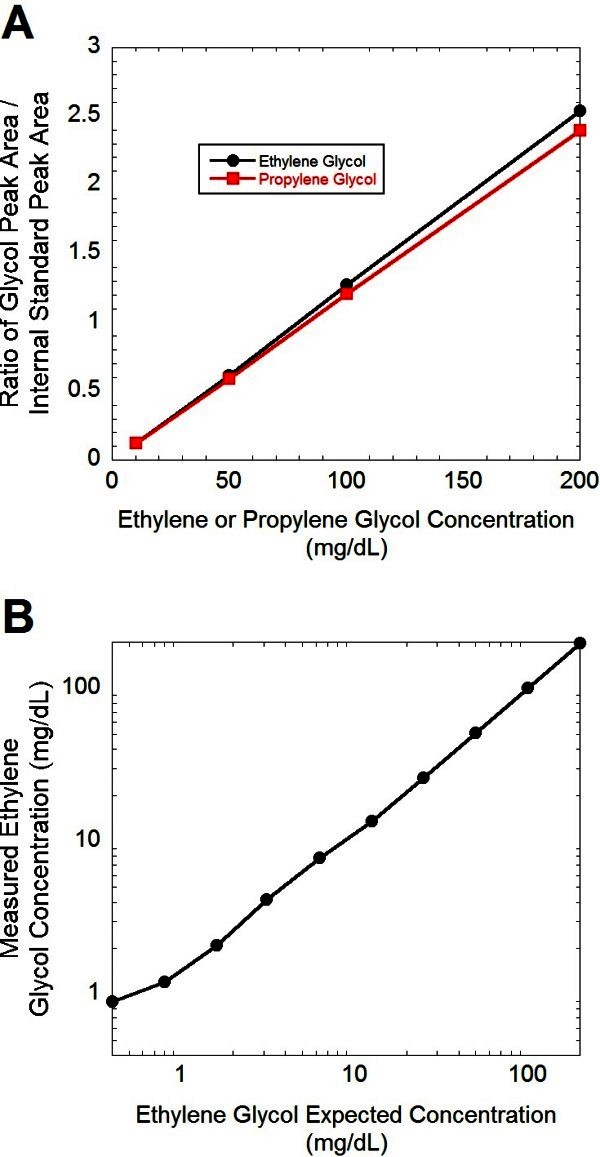
**Linearity plots of HS-GC analysis of ethylene glycol and propylene glycol.A** Linearity of ethylene glycol and propylene glycol shown using linear y-axis. **B** Linearity of ethylene glycol using a logarithmic y-axis for broader view of linearity of method for measurement of ethylene glycol concentrations.

Under the described conditions, the retention times of ethylene glycol, propylene glycol and 1,3-propanediol (internal standard) were 2.45, 2.52, and 3.05 minutes, respectively (Figure 1). This method was linear from a range of 1 mg/dL to 200 mg/dL for ethylene glycol and 10 mg/dL to 200 mg/dL for propylene glycol (Figure 2), with a correlation of r^2^ = 0.99 achievable for both ethylene glycol and propylene glycol. It is well established that ethylene glycol plasma concentrations do not always correlate with clinical severity (Porter 2012), with cases of severe toxicity described with ethylene glycol in the 5–10 mg/dL range (Rosano et al. 2009; Moreau et al. 1998; Porter et al. 2001). Patients that present many hours after ingestion are at particular risk for severe toxicity and renal damage (Porter 2012). Intra and inter-day precision and accuracy at ethylene glycol and propylene glycol concentrations of 25 mg/dL are summarized in Table 1.

**Table 1:** **Accuracy and precision studies**

	Ethylene glycol	Propylene glycol
**Nominal concentration (mg/dL)**	25	25
**Intra-day mean (n = 5)**	25.9 ± 0.4	25.8 ± 0.2
**Intra-day CV**	1.47%	0.86%
**Inter-day (n = 15) mean**	25.8 ± 0.3	25.7 ± 0.3
**Inter-day CV**	1.16%	1.32%

No interference was detected from 2,3-butanediol, diethylene glycol (Williams et al. 2000), or from patients with elevated levels of ethanol. Numerous samples from patients presenting to the emergency room (the typical patient population requiring rapid glycol analysis) were tested to check for interfering peaks, with none detected. Serum and plasma specimens were both acceptable, including from EDTA anticoagulated tubes and plasma separator tubes using lithium heparin as anticoagulant.

### Retrospective analysis of ethylene glycol ingestions

In a retrospective analysis of samples analyzed at the hospital core Clinical Chemistry laboratory, a total of 36 patients were found to have detectable ethylene glycol in plasma/serum. Three patients presented with two separate admissions for ethylene glycol toxicity. A summary of these patients is found in Additional file 1. There was only one fatality. This occurred in a patient with an estimated ingestion of 1 gallon of antifreeze who presented with a plasma ethylene glycol concentration of 1282 mg/dL. Two patients survived ingestions that resulted in ethylene glycol concentrations exceeding 800 mg/dL; one of these patients was in coma for 7 days and ultimately spent 33 days on an inpatient unit prior to discharge. Thirty-five patients had laboratory studies sufficient to calculate both osmolal and anion gaps. Of these 35 patients, 13 had both osmolal and anion gaps, 13 had only an osmolal gap, 8 had only an anion gap, and 1 had neither an osmolal nor anion gap.

All but two ingestions were deliberate self-harm attempts. Of the two non-self-harm attempts, one was an ingestion based on someone else passing antifreeze off as an ethanolic drink; the other was a teenager ingesting antifreeze in a misguided effort to achieve intoxication. Arterial blood gas analysis was performed in 25 cases; acidosis (pH < 7.35) was seen in 13 of these cases. Ethanol was detected in 13 of 36 cases, in 3 cases with plasma ethanol concentrations exceeding 200 mg/dL. It is likely in some cases that prior consumption of ethanol provided some degree of protection from ethylene glycol ingestion due to inhibition of metabolism by alcohol dehydrogenase. Propylene glycol was detected in 5 cases (discussed in more detail below).

### Detection of propylene glycol in patient samples

We detected propylene glycol at a plasma concentration of 10.0 mg/dL or greater in a total of 30 patients (37 total measurements). The most common presentation in these patients was drug overdose or toxic ingestion including acetaminophen overdose (6 patients), ethylene glycol ingestion (6 patients), prescription drug overdose (4 patients), and ingestion of unknown substances (3 patients). Five patients presented with suspected ethanol withdrawal. A summary of these patients is found in Additional file 2. Chart review identified likely iatrogenic sources of propylene glycol in 22 patients (intravenous lorazepam – 15 patients; intravenous diazepam – 1 patient; activated charcoal – 6 patients). Additional file 2 also indicates the estimated contribution of the plasma propylene glycol to the osmolal gap, using the propylene glycol concentration (in mg/dL) divided by 7.2 (Zar et al. 2007). In six of the patients, the estimated contribution of propylene glycol to the osmolal gap was necessary in reaching an osmolal gap cutoff of 15, which was the level of osmolal gap deemed abnormal at the medical center.

### Implications for toxicology analysis

The HS-GC method presented here provides rapid quantitation of ethylene glycol and propylene glycol in human plasma or serum. The instrumentation is the same as that used for HS-GC measurements of ethanol and ‘toxic alcohols’ such as methanol and isopropanol, allowing for use of a single HS-GC analyzer for determination of plasma concentrations of toxic alcohols and glycols. HS-GC analysis of the glycols, even following derivitization, does require higher headspace temperature (140°C) due to the lower vapor pressure and higher boiling points of the phenylboronic derivatives of ethylene and propylene glycol compared to the alcohols. HS-GC of underivitized ethylene and propylene glycol is likely possible but would require headspace temperatures higher than typically possible for most commercial headspace units.

### Clinical importance of propylene glycol

The results presented here indicate that detection of propylene glycol is common in hospital patients, particularly those managed from drug overdoses or other toxic ingestions. The major source of propylene glycol comes from iatrogenic administration of poorly water-soluble medications, especially intravenous lorazepam used for sedation. Intravenous formulations of lorazepam may contain up to 80% (v/v) propylene glycol. Other intravenous medications that may contain propylene glycol include diazepam, etomidate, and phenytoin (Al-Khafaji et al. 2002; Arbour 1999; Chicella et al. 2002; Parker et al. 2002). Another source of propylene glycol is activated charcoal preparations that use propylene glycol as the excipient to make the charcoal less gritty and easier to administer (Krasowski et al. 2012). Although the toxic plasma concentration of propylene glycol is not well-defined, toxicity has been reported with plasma concentrations as low as 100 mg/dL (Zar et al. 2007). However, even if not directly toxic, propylene glycol increases plasma osmolality and complicates the use of osmolal gap in clinical diagnosis and management, especially for patients receiving multiple doses of propylene glycol-containing medication, as may be done in intubated patients requiring extended sedation (Krasowski et al. 2012). Propylene glycol has also been reported as an interferent in enzyme assays for ethylene glycol (Malandain & Cano 1996; Juenke et al. 2011). Our study demonstrates that propylene glycol may be detected in patients who have ingested ethylene glycol, with iatrogenic drugs such as intravenous lorazepam or activated charcoal being common sources of propylene glycol. These findings illustrate the importance of specific assays for determination of ethylene glycol in human samples.

### Clinical importance of chromatographic methods for ethylene glycol

The retrospective study performed illustrates the importance of having a specific method for ethylene glycol. The majority of cases had either an osmolal gap or anion gap or both; however, in a number of patients, these gaps were only slightly elevated and 1 patient presented without either an osmolal or anion gap. In addition, 13 cases presented with co-ingestion of ethanol. Intravenous ethanol was used as treatment in 8 cases as well. Chromatographic analysis thus continues to play important role in detecting and managing ethylene glycol intoxications.

## Electronic supplementary material

Additional file 1: **Clinical history and laboratory data on patients with ethylene glycol ingestions.** (XLS 34 KB)

Additional file 2: **Clinical history and laboratory data on patients with propylene glycol plasma concentration of 10 mg/dl or greater.** (XLS 38 KB)

## References

[CR1] Al-Khafaji AH, Dewhirst WE, Manning HL (2002). Propylene glycol toxicity associated with lorazepam infusion in a patient receiving continuous veno-venous hemofiltration with dialysis. Anesth Analg.

[CR2] Almaghamsi AM, Yeung CK (1997). Osmolal gap in alcoholic ketoacidosis. Clin Nephrol.

[CR3] Arbour RB (1999). Propylene glycol toxicity related to high-dose lorazepam infusion: case report and discussion. Am J Crit Care.

[CR4] Balikova M, Kohlicek J (1988). Rapid determination of ethylene glycol at toxic levels in serum and urine. J Chromatogr.

[CR5] Barceloux DG, Krenzelok EP, Olson K, Watson W (1999). American Academy of Clinical Toxicology Practice Guidelines on the Treatment of Ethylene Glycol Poisoning. Ad Hoc Committee. J Toxicol Clin Toxicol.

[CR6] Braden GL, Strayhorn CH, Germain MJ, Mulhern JG, Skutches CL (1993). Increased osmolal gap in alcoholic acidosis. Arch Intern Med.

[CR7] Brooks DE, Wallace KL (2002). Acute propylene glycol ingestion. J Toxicol Clin Toxicol.

[CR8] Chicella M, Jansen P, Parthiban A, Marlowe KF, Bencsath FA, Krueger KP, Boerth R (2002). Propylene glycol accumulation associated with continuous infusion of lorazepam in pediatric intensive care patients. Crit Care Med.

[CR9] Dursun H, Noyan A, Cengiz N, Attila G, Buyukcelik M, Soran M, Seydaoglu G, Bayazit AK, Anarat A (2007). Changes in osmolal gap and osmolality in children with chronic and end-stage renal failure. Nephron Physiol.

[CR10] Garcia-Morales EJ, Cariappa R, Parvin CA, Scott MG, Diringer MN (2004). Osmole gap in neurologic-neurosurgical intensive care unit: Its normal value, calculation, and relationship with mannitol serum concentrations. Crit Care Med.

[CR11] Gill GV, Osypiw JC, Shearer E, English PJ, Watson ID (2005). Critical illness with hyponatraemia and impaired cell membrane integrity--the "sick cell syndrome" revisited. Clin Biochem.

[CR12] Guglielminotti J, Pernet P, Maury E, Alzieu M, Vaubourdolle M, Guidet B, Offenstadt G (2002). Osmolar gap hyponatremia in critically ill patients: evidence for the sick cell syndrome?. Crit Care Med.

[CR13] Houze P, Chaussard J, Harry P, Pays M (1993). Simultaneous determination of ethylene glycol, propylene glycol, 1,3-butylene glycol and 2,3-butylene glycol in human serum and urine by wide-bore column gas chromatography. J Chromatogr.

[CR14] Huff JS (1990). Acute mannitol intoxication in a patient with normal renal function. Am J Emerg Med.

[CR15] Jammalamadaka D, Raissi S (2010). Ethylene glycol, methanol and isopropyl alcohol intoxication. Am J Med Sci.

[CR16] Juenke JM, Hardy L, McMillin GA, Horowitz GL (2011). Rapid and specific quantification of ethylene glycol levels: adaptation of a commercial enzymatic assay to automated chemistry analyzers. Am J Clin Pathol.

[CR17] Khajuria A, Krahn J (2005). Osmolality revisited--deriving and validating the best formula for calculated osmolality. Clin Biochem.

[CR18] Krasowski MD, Wilcoxon RM, Miron J (2012). A retrospective analysis of glycol and toxic alcohol ingestion: utility of anion and osmolal gaps. BMC Clin Pathol.

[CR19] Kraut JA, Kurtz I (2008). Toxic alcohol ingestions: clinical features, diagnosis, and management. Clin J Am Soc Nephrol.

[CR20] Lynd LD, Richardson KJ, Purssell RA, Abu-Laban RB, Brubacher JR, Lepik KJ, Sivilotti ML (2008). An evaluation of the osmole gap as a screening test for toxic alcohol poisoning. BMC Emerg Med.

[CR21] Malandain H, Cano Y (1996). Interferences of glycerol, propylene glycol, and other diols in the enzymatic assay of ethylene glycol. Eur J Clin Chem Clin Biochem.

[CR22] Moreau CL, Kerns W, Tomaszewski CA, McMartin KE, Rose SR, Ford MD, Brent J (1998). Glycolate kinetics and hemodialysis clearance in ethylene glycol poisoning. META Study Group. J Toxicol Clin Toxicol.

[CR23] Parker MG, Fraser GL, Watson DM, Riker RR (2002). Removal of propylene glycol and correction of increased osmolar gap by hemodialysis in a patient on high dose lorazepam infusion therapy. Intensive Care Med.

[CR24] Porter WH (2012). Ethylene glycol poisoning: quintessential clinical toxicology; analytical conundrum. Clin Chim Acta.

[CR25] Porter WH, Auansakul A (1982). Gas-chromatographic determination of ethylene glycol in serum. Clin Chem.

[CR26] Porter WH, Rutter PW (2010). Improved GC-MS procedure for simultaneous measurement of ethylene glycol and glycolic acid. Clin Chem.

[CR27] Porter WH, Rutter PW, Bush BA, Pappas AA, Dunnington JE (2001). Ethylene glycol toxicity: the role of serum glycolic acid in hemodialysis. J Toxicol Clin Toxicol.

[CR28] Rosano TG, Swift TA, Kranick CJ, Sikirica M (2009). Ethylene glycol and glycolic acid in postmortem blood from fatal poisonings. J Anal Toxicol.

[CR29] Sklar AH, Linas SL (1983). The osmolal gap in renal failure. Ann Intern Med.

[CR30] Smith NB (1984). Determination of serum ethylene glycol by capillary gas chromatography. Clin Chim Acta.

[CR31] Williams RH, Shah SM, Maggiore JA, Erickson TB (2000). Simultaneous detection and quantitation of diethylene glycol, ethylene glycol, and the toxic alcohols in serum using capillary column gas chromatography. J Anal Toxicol.

[CR32] Wilson KC, Reardon C, Theodore AC, Farber HW (2005). Propylene glycol toxicity: a severe iatrogenic illness in ICU patients receiving IV benzodiazepines: a case series and prospective, observational pilot study. Chest.

[CR33] Wu AH, Kelly T, McKay C, Ostheimer D, Forte E, Hill D (1995). Definitive identification of an exceptionally high methanol concentration in an intoxication of a surviving infant: methanol metabolism by first-order elimination kinetics. J Forensic Sci.

[CR34] Zar T, Graeber C, Perazella MA (2007). Recognition, treatment, and prevention of propylene glycol toxicity. Semin Dial.

